# Towards region-specific propagation of protein functions

**DOI:** 10.1093/bioinformatics/bty834

**Published:** 2018-10-09

**Authors:** Da Chen Emily Koo, Richard Bonneau

**Affiliations:** 1 Department of Biology, Center for Genomics and Systems Biology, New York University, New York, NY, USA; 2 Center for Computational Biology, Flatiron Institute, Simons Foundation, New York, NY, USA; 3 Center for Data Science, New York University, New York, NY, USA

## Abstract

**Motivation:**

Due to the nature of experimental annotation, most protein function prediction methods operate at the protein-level, where functions are assigned to full-length proteins based on overall similarities. However, most proteins function by interacting with other proteins or molecules, and many functional associations should be limited to specific regions rather than the entire protein length. Most domain-centric function prediction methods depend on accurate domain family assignments to infer relationships between domains and functions, with regions that are unassigned to a known domain-family left out of functional evaluation. Given the abundance of residue-level annotations currently available, we present a function prediction methodology that automatically infers function labels of specific protein regions using protein-level annotations and multiple types of region-specific features.

**Results:**

We apply this method to local features obtained from InterPro, UniProtKB and amino acid sequences and show that this method improves both the accuracy and region-specificity of protein function transfer and prediction. We compare region-level predictive performance of our method against that of a whole-protein baseline method using proteins with structurally verified binding sites and also compare protein-level temporal holdout predictive performances to expand the variety and specificity of GO terms we could evaluate. Our results can also serve as a starting point to categorize GO terms into region-specific and whole-protein terms and select prediction methods for different classes of GO terms.

**Availability and implementation:**

The code and features are freely available at: https://github.com/ek1203/rsfp.

**Supplementary information:**

Supplementary data are available at *Bioinformatics* online.

## 1 Introduction

Proteins are involved in nearly every cellular process and function, including cell organization, biochemical catalysis, signaling and transport. The exponentially large number of possible sequence combinations enables proteins to exhibit the necessary diverse sequential, structural and functional properties required by the cell. Protein function is often defined or modified by specific interactions with other molecules, therefore knowing what, where and how proteins interact is important in elucidating the cellular machinery of life (Alberts *et al.*, 2002). Proteins can be multifunctional by having completely different functions in different contexts [‘moonlighting proteins’ like crystallins (Jeffery, 2009; Piatigorsky, 1998)], by binding to multiple substrates and catalyzing multiple reactions [‘promiscuous proteins’ (Hult and Berglund, 2007; Khersonsky and Tawfik, 2010)] or by having combinations of domains in different sequential orders (Bashton and Chothia, 2002). Comprehensive experimental characterization of protein function is thus a laborious, expensive and time-consuming process, which is especially true for proteins found in non-model and multicellular organisms.

With the advent of the Gene Ontology (GO) project (Ashburner *et al.*, 2000), computational prediction of protein function has become more viable and it is an on-going quest to improve the number and quality of predictions. Many different approaches have been proposed over decades (Cozzetto *et al.*, 2013, 2016; Jensen *et al.*, 2002; Lanckriet *et al.*, 2004; Martin *et al.*, 2004; Mostafavi *et al.*, 2008; Roy *et al.*, 2010; Sharan *et al.*, 2007; Troyanskaya *et al.*, 2003) and reviewed extensively (Bernardes and Pedreira, 2013; Bork *et al.*, 1998; Kihara, 2016; Lee *et al.*, 2007; Rost *et al.*, 2003).

Although not an all-encompassing evaluation, results from large-scale Critical Assessment of protein Function Annotation (CAFA) experiments (Jiang *et al.*, 2016; Radivojac *et al.*, 2013) and related blind tests like MouseFunc (Peña-Castillo *et al.*, 2008) offer important insights into the performances of state-of-the-art computational protein function prediction methods.

The underlying principle of protein function prediction is the transfer of function from a known protein to a query protein based on shared features. Such features include protein sequence, structure and pairwise associations from high-throughput experimental data like protein–protein interaction and gene co-expression. BLAST (Altschul *et al.*, 1990), for example, is one of the most widely used sequence-based tools and is regularly used as a benchmark to evaluate the performances of more complex methods. Due to the nature of experimental annotation (and the prevalence of genetics as a means of connecting genes to functions), most protein function prediction methods operate at the whole-protein level (i.e. functions are transferred directly between whole-chain proteins). However, the majority of proteins are composed of one or more structural and functional units called domains (Gerstein, 1998), which can function independently or in combination with other domains [‘supra-domains’ (Vogel *et al.*, 2004)].

Ideally, functions wholly encapsulated within such regions should be confined and uncoupled from other regions of the protein in a region-specific annotation scheme. This is especially true for GO terms in the Molecular Function (MF) branch of the ontology. Currently, a few such annotated domain-centric resources exist through a combination of manual and automated curation [e.g. Pfam (Finn *et al.*, 2016b), SUPERFAMILY (Gough *et al.*, 2001), CATH-GENE3D (Sillitoe and Furnham, 2016), metadatabase InterPro (Finn *et al.*, 2016a)], allowing functions to be transferred to novel proteins assigned with known and annotated domain families. Other domain-centric methods have also been described previously (Cozzetto *et al.*, 2016; Das *et al.*, 2015; Fang and Gough, 2013; Forslund and Sonnhammer, 2008; Lopez and Pazos, 2013; Rentzsch and Orengo, 2013; Schug *et al.*, 2002) to automatically associate functions directly to domain families before integrating them for protein function prediction.

These resources are generally very sparse as they require a fine balance between sufficient coverage of the domain space and the applicability of the annotations to all proteins matching the given domain signature (Burge *et al.*, 2012). This is especially problematic for large and diverse families, and hinders the mapping of specific GO terms. Another common weakness of these domain-centric approaches is that they depend entirely on predicted domain family assignments, which not only differ based on different classification and identification schemes, but are also constantly changing and updating (Sangrador-Vegas *et al.*, 2016). Additionally, these predicted assignments only cover about a third of the total residues in the proteomes. For example, even though approximately 55% of yeast and 68% of human protein sequences [UniProtKB (Bateman *et al.*, 2017) reference proteomes release 2017_10] have at least one ‘DOMAIN’ entry type assigned [InterPro database (Finn *et al.*, 2016a) release 65.0, Oct 2017], less than 32 and 38% of total residues in each proteome, respectively, are actually covered by the assignments. This can be due to the fact that the majority of domain families identified are structured domains. Intrinsically disordered regions, which are prevalent in eukaryotic genomes and have been established to actively participate in diverse protein functions (Van Der Lee *et al.*, 2014), are excluded entirely from functional evaluation. In addition, the treatment of domains as binary features of proteins prevent the transfer of function from annotated to unannotated domain families, which can have shared functions as well.

**Fig. 1.**
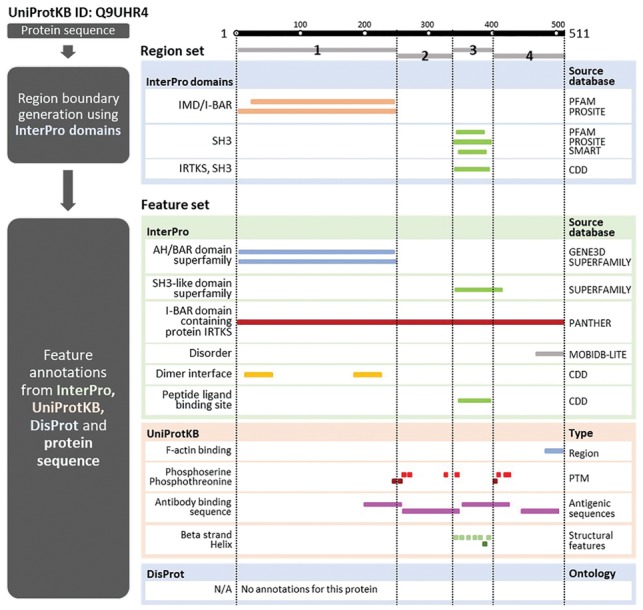
Diagram of processing pipeline using ‘BAI1-associated protein 2-like protein 1’ (*BAIAP2L1*, UniProtKB accession: Q9UHR4) as an example. Region boundaries are delineated by vertical lines based on consensus domain assignments from different InterPro member databases. Below, features assigned to the protein sequence are grouped by source databases for clarity

Therefore, we find it pertinent to decompose proteins into ‘**regions’**, which are *continuous* sections of protein sequences partitioned by consensus assignments from InterProScan to ensure full sequence coverage (see Section 2.1 for more details). This allows us to then build a method that can transfer protein labels at the region level explicitly, including regions that are not covered by traditional domain assignments. For example in Figure 1, instead of representing ‘BAI1-associated protein 2-like protein 1’ (*BAIAP2L1*, UniProtKB accession: Q9UHR4) as a 511 residue protein with two domains, an IMD/I-BAR domain (residues 1–249) and an SH3 domain (residues 339–402), we will represent this protein as four regions, with regions 1 and 3 containing the well-annotated domains and the remaining regions 2 (residues 250–338) and 4 (residues 403–511) containing (prior to prediction) no assigned domain families. These unassigned regions will still contain sequence information (and sequence derived features) and other site-specific feature annotations, such as post-translational modifications and F-actin binding sites, from databases with manual curation like UniProtKB (Bateman *et al.*, 2017) that can provide functional clues (features).

Here, we detail our approach to generating protein regions using curated site-specific features and to localizing known protein function labels to these regions automatically based on related approaches to structured sentiment analysis (Kotzias *et al.*, 2015). We evaluate the prediction accuracy of our region-specific framework for a variety of GO terms at both region- and protein-levels for experimentally annotated and reviewed protein sequences from Swiss-Prot and describe performance improvements over using a whole-protein baseline model. Our results show that many GO terms benefit from applying this region-specific framework and that different types of GO terms should be treated differently in function prediction pipelines depending on their extent of functional localization.

## 2 Materials and methods

The general process outline of our method is to: (i) split protein sequences into potential functional regions based on the presence and absence of domain and protein family assignments, (ii) encode the regions as separate feature vectors based on data sources summarized in Table 1 and (iii) train model to infer region function labels from known protein labels. This is summarized in Figure 1. The taxonomic composition of the protein dataset can be found in Supplementary Figure S1.

**Table 1.** Data sources

Data type	Version
Protein set	UniProtKB Reference Proteomes release 2018_02
GO annotations (protein)	UniProt-GOA release 155 (2016-05) and 175 (2018-02) [non-IEA only^a^]
GO annotations (domain/region)	external2go (2016-05-28 and 2018-03-01), inferred from binding sites (NBench and BioLiP)
InterPro features	InterProScan 5.28-67.0
UniProt features	UniProtKB/Swiss-Prot Release 2018_02
DisProt features	DisProt 7 release 0.5

* Annotations with the following evidence codes ‘EXP’, ‘IDA’, ‘IMP’, ‘IGI’, ‘IEP’, ‘TAS’, ‘IC’, ‘IPI’.

### 2.1 Generating region boundaries

The first step in this approach is to split the protein sequences into potential functional regions. To do that, we processed the amino acid sequences with InterProScan (Jones *et al.*, 2014) and used the feature annotations to build consensus region boundaries. InterPro entry types used include Domain, Family, Homologous superfamily and select unintegrated signatures from InterPro (Finn *et al.*, 2016a), Signal peptide, Transmembrane and Non-transmembrane from PHOBIUS (Käll *et al.*, 2004) and Disorder from MobiDB-lite (Necci *et al.*, 2017). All Repeats and Sites annotations are excluded during this process. Aside from Signal peptide, which is included whenever available, initial region boundaries are assigned exclusively by types in the following order of precedence: Domain, Family and Homologous superfamily, Unintegrated signatures, Transmembrane, Non-transmembrane and Disorder.

Unassigned **terminal regions** less than 18 residues in length [selected based on signal length distributions (Bendtsen *et al.*, 2004)] are merged with immediate neighboring regions to remove excessive numbers of short peptides without losing potential targeting and retention signal peptides, while longer terminal regions are retained as separate regions. **Inter-domain regions** less than 20 residues in length [selected based on linker length distribution (Chen *et al.*, 2013)] are also discarded to remove the majority of linker regions.

On average, each protein has a mean of 3.14 and a median of 3 regions. The complete distribution of regions per protein can be found in Supplementary Figure S2.

### 2.2 Data representation and feature generation

Each protein/region is represented as a fixed-length vector in four different feature spaces encoded with the data types listed below.

1. ***K*-mers**—a collection of consecutive, overlapping, *k*-residue long sub-sequences of the sequence of the region itself. For example, a sequence of ‘ABCDE’ will result in 3-mers of ‘ABC’, ‘BCD’ and ‘CDE’. The length of 3 was tested to get a compromise between capturing sufficient protein fold information and having a feature vector that is not too large to deal with.
2. **Keywords**—a vocabulary of individual words parsed from descriptions of features assigned from UniProtKB, InterPro entries, original member databases and DisProt. For example, a region assigned with feature ‘WD repeat-containing’ will contain keywords ‘WD’, ‘repeat’ and ‘containing’, allowing the region to have a non-zero similarity score when compared to another that is assigned with ‘WD repeat’ (i.e. ‘WD’ and ‘repeat’). In addition, this allows us to aggregate features from the different databases into a homogeneous feature space.

For further comparisons, the InterPro entry IDs and signature IDs assigned to the regions (at least 75% overlap) are used directly as they require no further processing.

3. **InterPro entry IDs**—a collection of unique IDs (which can map to protein families, domain families, repeats, sites), assigned to the regions by InterPro.
4. **Signature IDs** from InterPro member databases—a collection of unique IDs (which can map to protein families, domain families, repeats, sites), assigned to the regions by the member databases of InterPro. This also includes unintegrated entries like signal peptide and transmembrane topology predictions from tools like Phobius (Käll *et al.*, 2004) through InterProScan (Finn *et al.*, 2016a).

The hypothesized advantage of using InterPro entry IDs is that the feature set would be concise and curated, whereas the advantage of using the underlying Signature IDs is that the feature set would be more sensitive to functional differences. This is due to the fact that different databases use different models and methods to classify and identify the assigned features, so not all regions containing the same InterPro IDs will be matched to the same set of signature IDs. For example, the ‘C2 domain’ (IPR000008) groups four contributing signatures and is assigned to both ‘Fer-1-like protein 5’ (A0AVI2) and ‘Extended synaptotagmin-3’ (A0FGR9) proteins. However, only three out of the four signatures matched ‘Fer-1-like protein 5’, while all four signatures matched ‘Extended synaptotagmin-3’.

The resulting frequency matrices for the features are then transformed into TF-IDF weights (Salton and McGill, 1986) (a well-established technique in Natural Language Processing) to upweight features that occur in fewer proteins and downweight features that occur in many.

The dimensions of the different feature types are shown in Supplementary Table S1 and a visual example of the differing pairwise similarity scores (cosine similarity) for a set of 500 regions can be viewed in Supplementary Figure S3. The number and fraction of proteins and regions containing each of the feature type are summarized in Table 2 and the number of regions without InterPro domains and families but are covered by Signature IDs and Keyword features are summarized in Supplementary Table S2.

**Table 2.** Feature coverage

Feature type	Counts (%)
**Protein**	73 224 (100%)
InterPro domains	50 641 (69.2%)
All InterPro entries (incl. sites)	70 662 (96.5%)
Signatures	72 441 (98.9%)
Keywords	73 157 (99.9%)
**Regions**	230 186 (100%)
InterPro domains	91 276 (39.7%)
All InterPro entries (incl. sites)	137 278 (59.6%)
Signatures	190 052 (82.6%)
Keywords	204 684 (88.9%)

### 2.3 Our region-specific cost function

To transfer known protein labels to the respective functional regions, we have extended an approach called Group-Instance Cost Function (GICF) (Kotzias *et al.*, 2015), initially applied to sentiment analysis of sentences within larger document and user hierarchies. Applied to protein function prediction, these prior works are analogous to identifying positive and negative labels (for a given GO term) for regions within proteins, given only known labels of whole proteins.

This method involves minimizing a cost function that penalizes differences in predicted region scores based on their pairwise feature similarities and also differences between predicted protein scores (aggregated from the constituent region scores) with known protein labels. Additional model components are introduced here to account for differences between predicted region scores with known domain labels (from manually curated databases) and to reduce over-fitting to training data (additional regularization terms have been added).

All together, our cost function consists of 4 terms, each of which enforces the following constraints respectively: (i) predicted region scores should aggregate to give the correct protein scores when evaluated against known protein labels—positive or negative (protein-level constraint), (ii) regions with similar features should have similar predicted scores, (iii) predicted region scores should agree with any known domain-level labels—only positive due to the sparsity (region-level constraints), (iv) model should not be overfitted to the training set, which is a standard procedure in machine learning but was missing from the original cost function (regularization term).

For each GO term, an independent *θ* is estimated from the following cost function:

(1) $$\begin{array}{c} J(\theta )=\frac{1}{N_{p}}\sum_{k=1}^{N_{p}}\Delta ({\hat{Y}}_{k},Y_{k})+\frac{w_{1}}{N_{r}^{2}}\sum_{i=1}^{N_{r}}\sum_{j=1}^{N_{r}}\kappa (x_{i},x_{j})\Delta ({\hat{y}}_{i},{\hat{y}}_{j})\\ +\frac{w_{2}}{N_{r+}}\sum_{i\subset r_{+}}\Delta ({\hat{y}}_{i},y_{i})+\frac{\lambda }{N_{p}}{\left\|\theta \right\|}_{2}^{2} \end{array}$$

where:

- $y_{i}\in \{0,1\}$ is the known label for region *i*,
- $Y_{k}\in \{0,1\}$ is the known label for protein *k*,
- ${\hat{Y}}_{k}=max({\hat{y}}_{i\subset r_{k}})\in [0,1]$ is the predicted score for protein *k*, obtained by getting the maximum predicted score of its containing regions *r_k_*,
- *N_r_* is the total number of regions, $N_{r+}$ is the number of positively annotated regions and $r_{+}$ is the subset of positively annotated regions across all proteins,
- *N_p_* is the total number of proteins,
- $\kappa (x_{i},x_{j})\in [0,1]$ is the similarity score between regions *x_i_* and *x_j_*, calculated using the cosine similarity of the feature vectors (pairwise scores below the 95th percentile—generated by randomly sampling 10,000 regions for each feature type—are set to 0),
- Δ is the square loss function, which is the square of the difference between the variables and
- *w_n_* and *λ* are the trained weights to balance the contributions of the different terms.

The cost function is based on the output of the following logistic regression model:

(2) $${\hat{y}}_{i}=\frac{1}{1+e^{-\theta^{T}x_{i}}}$$

where:

- *x_i_* is the input feature vector for region *i*,
- *θ* is the weight vector of the different contributing features and
- ${\hat{y}}_{i}\in [0,1]$ is the predicted score for region *i*.

The choice of logistic regression is due to the binary nature of individual labels (a given protein has the function or it does not) and the resulting probabilistic output that is useful for ranking the predictions. The seed (initial) *θ* was generated for each GO term by fitting a logistic regression model using protein-level features and known protein labels of the same training set. We used minibatch stochastic gradient descent with momentum to train the model. Values of all the hyperparameters are detailed in Supplementary Section 4.

### 2.4 Modes of evaluation

Non-IEA (Inferred from Electronic Annotation) annotations at two time-points were used to train and validate/test the models respectively. This ensures a more ‘realistic’ approach compared to cross-validation as only proteins with older annotations are used for training, while proteins that gained new annotations are used for validation and testing, allowing for a fully blind test.

For model training, protein-level annotations were obtained from UniProt-GOA and domain-level annotations were obtained from external2GO (see Table 1). Known domain-level annotations were propagated up to the parent proteins for consistency. For validation and testing, only non-IEA protein-level annotations were used. The details of the protein-level temporal holdout datasets can be found in Supplementary Section 3.2.

#### 2.4.1 Region-level evaluation

Due to their sparsity of domain-level annotations and the lack of region-level annotations, there is a lack of gold standard datasets that we can use as a benchmark to effectively evaluate many of the GO terms at the region-specific level. However, to show that this approach can successfully localize GO term labels to the correct regions, we decided to use binding site annotations from the following databases to evaluate their respective ligand binding GO terms: NBench (Miao and Westhof, 2015), as a source of nucleic acid binding sites for ‘DNA binding’ (GO: 0003677) and ‘RNA binding’ (GO: 0003723), and BioLiP (Yang *et al.*, 2013), as a source of magnesium and zinc ion binding sites for ‘magnesium ion binding’ (GO: 0000287) and ‘zinc ion binding’ (GO: 0008270). Although they may contain some errors, both databases are semi-manually curated from protein structures extracted from the Protein Data Bank.

Here, nucleic acid binding regions were defined as regions with more than 3 amino acid residues within a cutoff distance of 6 Å from a nucleic acid molecule in the complex to reduce spurious associations. However, as most proteins do not have structural data for the entire protein length, only regions with at least 80% structural coverage were considered in the evaluation as either a positive or negative example. The same training and validation proteins as the temporal holdout set were used for model selection.

Area Under Precision-Recall (AUPR) performances were generated for regions with structural coverage and were evaluated for each GO term individually. The method is then compared with a whole-protein baseline method (see Section 2.4.3) using two-tailed Wilcoxon signed rank test after 1000 rounds of bootstrapping. The details of the region-level datasets are found in Supplementary Section 3.1.

#### 2.4.2 Protein-level evaluation

To test if localizing GO term labels to their respective regions can improve overall function prediction at the protein level and if so, for which GO terms, we also conducted protein-level evaluations for all 67 MF-GO terms that meet the temporal holdout criteria. The predicted protein scores are obtained by getting the maximum predicted score of its containing regions.

#### 2.4.3 Baseline methods

BLAST predictions for regions and proteins were generated using the maximum pairwise sequence identity between the region/protein template and the protein targets [as used in CAFA Jiang *et al.* (2016)].

Logistic regression [implemented using source code from (Rebello, 2013)] was used as the whole-protein **baseline method** for comparison. It is trained directly on features from *whole proteins* (i.e. features from all regions plus those that span multiple regions) with regularization weights (*λ*) ranging from 0.1 to 100 and the same set of protein-level annotations, with no knowledge of region boundaries at all. The best estimated *θ_base_* based on the validation set was also used as the seed input *θ* to our cost function. This would give us an estimate of how well the predictions would do without the constraints of the region-specific framework. As the feature vectors are the same dimensions for regions and proteins, the *θ_base_* trained on whole proteins can be used to predict for both regions and proteins directly, allowing us to compare the scores predicted at the region- and protein-level.

## 3 Results and discussion

We show the ability of our method to localize binding labels to specific regions within proteins by comparing the predictions directly to binding sites extracted from protein-ligand structures. We also show that the added region-specific framework can lead to improvements in protein-level function predictions for many of the MF-GO terms that we tested in the section after.

### 3.1 Region-specific localization of binding terms

**Fig. 2.**
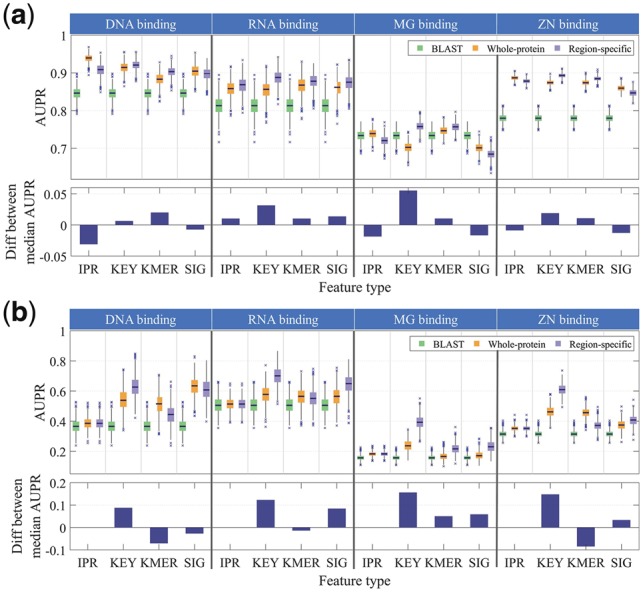
Performance comparisons of region-level predictions using BLAST (green), whole-protein baseline model (orange) versus our region-specific model (purple) for DNA (GO: 0003677), RNA (GO: 0003723), MG (GO: 0000287) and ZN (GO: 0008270) binding GO terms. IPR = InterPro IDs, KEY = Keywords, KMER = K-mers, SIG = Signature IDs. (**a**) Regions with InterPro assignments, (**b**) Regions without any InterPro assignments. **Upper panels:** Box plots showing the first quartile (Q1), median, third quartile (Q3) and outliers of AUPRs generated over 1000 rounds of bootstrapping. **Bottom panels:** Differences between the median AUPR between whole-protein baseline and region-specific methods corresponding to the pair of box plots directly above. Positive values indicate that the region-specific method outperforms the baseline and vice versa for negative values. Aside from the IPR performances in (**b**), all differences are significant to at least.001 level based on test statistics from two-tailed Wilcoxon signed rank test

Here, we detail tests of our method over a subset of GO terms that can be tied directly to protein sequence via structure, focusing on cases where protein structure analysis provides unambiguous localizations to proteins with residue-level resolution. Results from the region-level evaluation for four binding GO terms and four feature sets are shown in Figure 2, with Figure 2(a) containing regions with InterPro assignments and Figure 2(b) containing regions without InterPro assignments. The evaluation results of all of these regions combined can be found in the Supplementary Section 5.1.

We include both the BLAST and whole-protein baseline performances for comparison, with the bottom panels showing the differences in performances between the median AUPRs of our region-specific method and whole-protein baseline. This head-to-head comparison allows us to eliminate the differences in raw performances based on lack of informative features or annotations, allowing us to focus entirely on the effectiveness of using this region-specific framework for this particular set of feature types and GO terms. The main difference between the models (aside from the region-specific constraints) is that the whole-protein baseline model uses a combination of all the features found within the regions plus additional protein-level features that span multiple regions, like protein families, which would not exist when the regions are looked at in isolation.

When we look at regions with InterPro assignments, **InterPro IDs** and **Signature IDs** do not benefit much from this approach and perform worse than baseline in most binding terms at the region-level, while **Keywords** and ***K*-mers** show significant improvements across binding terms. This is not surprising given that both features can be found in regions without domain family assignments and are more discriminating within regions compared to **InterPro IDs** and **Signature IDs**, allowing for greater propagation of labels between regions.

On the other hand, when we look at regions without InterPro assignments (and thus cannot be predicted using **InterPro IDs**—the box plots show the performance when all scores are 0), **Keywords** and **Signature IDs** both show significant improvements across binding terms, while ***K*-mers** show decreases in performances. This suggests that there are informative features that exist in other databases that have yet to be incorporated into InterPro and that these regions likely have less sequence homology to the currently annotated proteins and regions.

Overall, **Signature IDs** show equivalent or better performance at the region-level compared to **InterPro IDs**, pointing to the value of unintegrated feature annotations, such as signal peptides and transmembrane helices, that would not be included in purely domain-centric methods. InterPro assignments are dominated by Domain and Family entry types (47 and 32% in yeast, 53 and 22% in human, respectively), and in the majority of cases, two regions are either assigned the same InterPro ID, and thus have a similarity score of 1, or are assigned different InterPro IDs, and thus have a similarity score of 0. As shown in Supplementary Figure S3, the resulting pairwise similarity values for **InterPro** are very sparse relative to the other feature types and that sparsity has the effect of reducing the effectiveness of term 2 in the cost function (equation 1), which encourages propagation of labels between regions.

In the future, it would be trivial to extend this method to other, perhaps more informative, sequence-derived representations like ProtVec (Asgari and Mofrad, 2015), biophysical properties of the amino acids themselves (Cozzetto *et al.*, 2016), or even structure-derived representations like contact maps and features extracted from known structures using the Rosetta energy function (Alford *et al.*, 2017) to take into account short- and long-range interactions between residues. In addition, we are also working on using autoencoders as a way to integrate the different feature types into a single, low-dimensional feature space.

#### 3.1.1 Structural examples of binding label localization

**Fig. 3.**
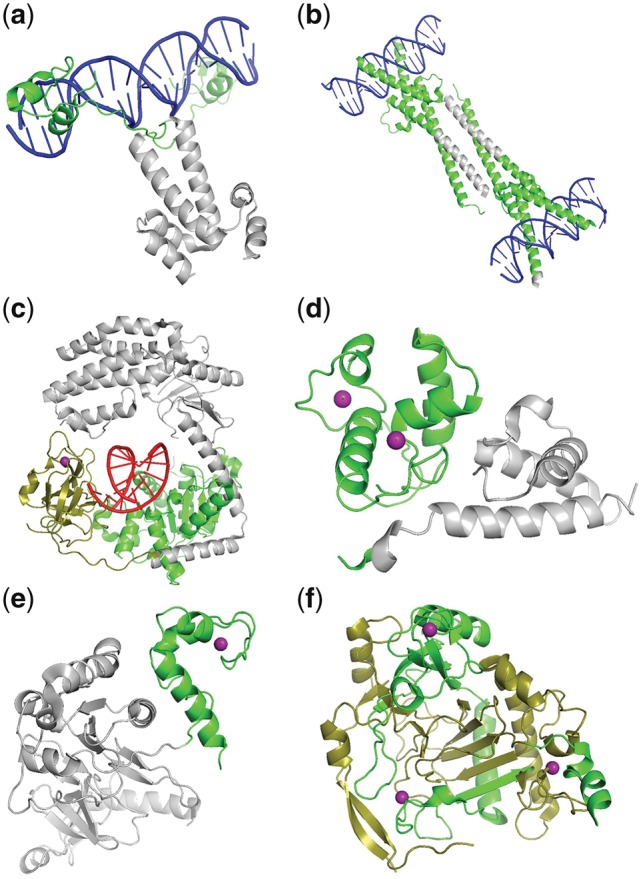
Structural examples of DNA (**a,b**), RNA (**c**) and Zn (**d–f**) binding predictions. Regions are colored in green if it is predicted to be positive for a given GO term. Different shades of green are used to delineate separate regions. The UniProtKB (PDB ids) are as follows: a) P04386 (3coq), b) P01106 (1nkp), c) O95786 (3zd6), d) P03081 (2pf4), e) P39286 (5uz4), f) Q6N021 (5deu). **Ligand colors:** DNA (blue). RNA (red), Zn (purple). Images are created with PyMOL (Schrödinger, LLC, 2015)

Here we leverage the binding proteins with known structures to investigate the performance and resolution of our method. Figure 3 shows some examples of the localizations of binding GO terms to specific regions within the protein using only **Keywords**. Regions are colored green according to positive predictions from Keyword features. We consider a region to be positive if its predicted score is larger than the threshold at which the F1 score (harmonic mean of precision and recall) is at its maximum for the given GO term. Different shades are used simply to delineate separate regions. None of these regions have been annotated at time of training or testing.

Figure 3a and b show crystal structures of proteins containing two chains with a DNA binding and a protein dimerization region. In both cases, our region-specific method successfully localized the DNA binding GO term only to the direct DNA binding regions, while excluding the dimerization regions. Figure 3c shows an example of RNA binding prediction for two neighbouring regions in space but not in sequence, while Figure 3d–f all highlight zinc-binding regions. In particular, the predicted binding region in Figure 3e does not have a domain assigned, while the regions in Figure 3f overlap two much larger regions outside of the available structural coverage. One is assigned as an oxygenase domain, while the other remains unassigned.

More details on the structures and binding residues can be found in Supplementary Section 5.2.

### 3.2 Expanded functional evaluation at protein level

After the initial proof of concept, which shows that our region-specific method can successfully localize binding GO term labels to specific regions without prior domain-level annotations, we extend the analysis to include GO terms from a larger number of functional categories by evaluating the predictions at the protein level.

**Fig. 4.**
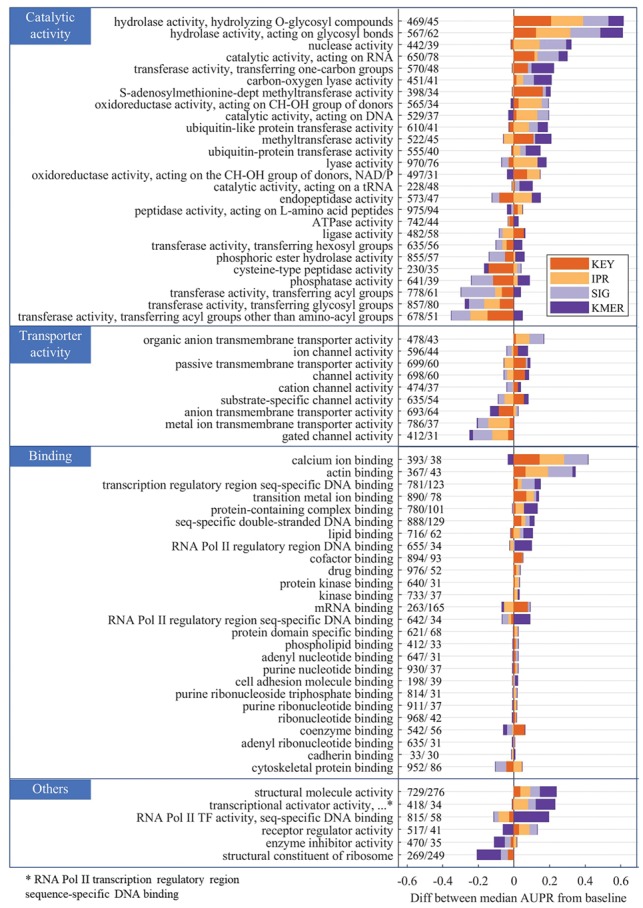
Stacked bar plots showing the differences in performance of protein-level predictions using the region-specific model versus the whole-protein baseline model using non-IEA annotations. KEY = Keywords, KMER = K-mers, IPR = InterPro IDs, SIG = Signature IDs. GO terms are grouped into the parent categories labeled in blue on the top left. The size of each block represents the difference between the median AUPR scores of our method and the baseline. Positive (right) means that our method performs better than the baseline and vice versa for negative (left). GO terms are sorted by the sum of the differences across features. Numbers next to each GO term = number of positive training and testing proteins. All differences shown are significant to at least .05 level

Figure 4 shows the differences in median AUPR performances of our region-specific method *relative to* the whole-protein baseline model using the same training, validation and test sets. Protein-level predictions over all four feature types are evaluated and all GO terms are grouped into the shared parental terms (shown in blue on the top left) for clarity, with isolated terms grouped together as ‘Others’. The differences are shown as colored bars stacked either in the positive (right) or negative (left) direction. Only differences that are significant (*P*-value < 0.05 level based on two-tailed Wilcoxon signed rank test) are shown on the plot. The numbers next to each GO term represent the number of positive training and testing proteins for that particular GO term (i.e. the particular class distributions during training and testing). Majority of the binding terms that do not show much difference from baseline are not predictable using any of the methods and this can be observed from the raw performances found in Supplementary Section 6.

Overall, our results here show that this framework can improve protein-level function predictions for many of the MF-GO terms evaluated, especially for Keywords and *K*-mer features that allow for better label propagation between regions. The results also suggest that the performance relative to the whole-protein baseline model can give us a way to characterize region-specificity of different GO terms and thus inform us on how to treat them during the function prediction process.

The majority of **Binding** terms show improved performance regardless of feature type, especially for terms with well-annotated binding site annotations from InterPro and UniProtKB like metal ion and DNA bindings. About two-thirds of the **Catalytic activity** GO terms evaluated also show better performance across features when the region-specific method is used, suggesting that it is possible and helpful to localize some GO terms to single regions. For those that perform worse, it is likely that specific features from multiple regions are needed for the association or if the protein family is composed of domains found in diverse proteins, such that a strong association can only be made at the level of the protein family itself.

Majority of the **Transporter activity** GO terms show a decrease in performance with InterPro and Signature IDs, but improved performance with Keywords, highlighting the advantage of using text descriptions directly for better label propagation between regions. For InterPro IDs in particular, if the association to the region features are weak in the training set (i.e. the fraction of proteins with those features that are positively annotated with that GO term is low), then term 2 of the cost function (Equation 1) will weaken the associations further due to high region similarity to regions in negatively annotated proteins (refer to a similar discussion in Section 3.1 on sparsity of InterPro IDs).

Cases where *K*-mers show large improvements in performance suggest that the GO term can be localized to sequence-specific regions. This is supported by the improvements in GO terms such as ‘RNA polymerase II regulatory region sequence-specific DNA binding’ (GO: 0000977) under **Binding and** ‘RNA polymerase II transcription factor activity, sequence-specific DNA binding’ (GO: 0000981) under **Others**.

## 4 Conclusion

In this work, we have described a function prediction pipeline to localize protein-level annotations to specific regions within the protein. It is based on the compact biologically reasonable assumption that functional homology is mediated by regions with similar (if unknown) features and is built around a cost function that takes into account regions with unassigned domain families but contain existing feature annotations.

The results from our region-level evaluation using ligand binding datasets show that our method can successfully localize functions known to be site-specific to their respective functional regions and performs significantly better than the whole-protein variant. We also evaluate the performance of our region-specific prediction method at the whole-protein level to determine the protein functions that benefit from our explicit region localization and find that, while localization improves performance for some functions, it also decreases performance for others.

This difference in the effect of mapping function to specific regions supports the notion that different GO terms have different levels of operational units and that they should be treated differently in protein function prediction pipelines to take that into account (with some functions tied to small active/binding regions, some tied to domains and some that need multiple domains and regions for proper functioning). Our results serve as a starting point to begin categorizing GO terms into region-specific and protein-wide sub groups to maximize the predictive performance of protein function prediction for each GO term and to provide a framework for selecting correct function prediction methods for different functions.

Future work would include introducing a hierarchy of region boundaries within a single protein to allow for different levels of label propagation for different GO terms, and also the use of different feature types simultaneously to consolidate different sources of information. One could, in principle, use autoencoders or NNMF to integrate the different feature types into a single, low-dimensional feature space that would be well-suited to our region-level model (Gligorijević and Pržulj, 2015; Li *et al.*, 2016). We will also experiment with combining our region-specific model with protein–protein network data using methods such as deepNF (Gligorijević *et al.*, 2018) to incorporate known overarching relationships between proteins for a more comprehensive function prediction tool.

## Supplementary Material

bty834_Supplementary_Data
