# Supplementary material for: Towards region-specific propagation of protein functions
Source: Bioinformatics. 2018 Oct 9;35(10):1737–44. doi: 10.1093/bioinformatics/bty834 (PMC6513163; doi:10.1093/bioinformatics/bty834)
Supplement: bty834_Supplementary_Data [file bty834_supplementary_data.pdf]

Supplementary Material
Towards region-specific propagation of protein functions

Da Chen Emily Koo *[1] and Richard Bonneau †[1,2,3]

[1]Department of Biology, Center for Genomics and Systems Biology, New York University, New York, NY 10003, USA
[2]Center for Computational Biology, Flatiron Institute, Simons Foundation, New York, NY 10010, USA
[3]Center for Data Science, New York University, New York, NY 10011, USA

*emily.koo@nyu.edu
†rb133@nyu.edu

1 Dataset

Figure S1: Taxonomic composition of protein dataset used.
Total number of proteins = 73,224

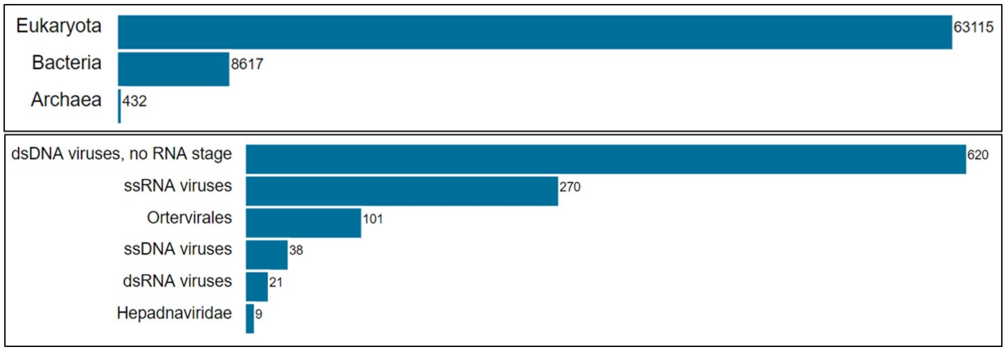

(a) Taxonomy view of protein set

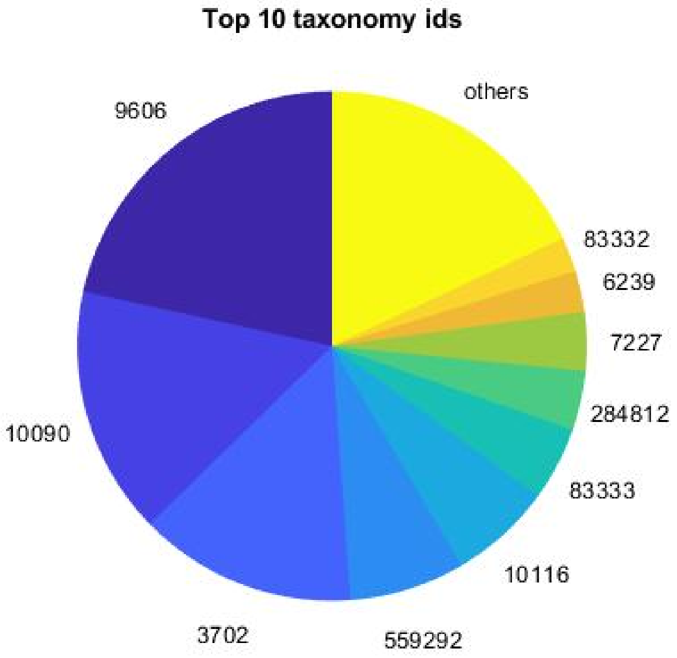

(b) Top 10 taxonomy ids

2 Feature representations

Figure S2: Distribution of the number of regions per protein. The solid red line represents the median (3) while the dotted red line represents the mean (3.14). 49 proteins containing more than 25 regions were excluded from this plot for clarity.

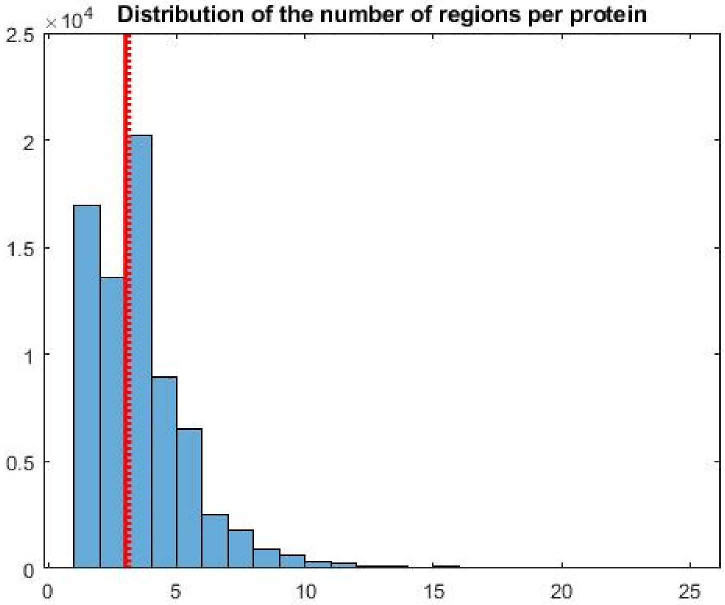

Table S1: Dimensions of features

K-mers	Keywords	InterPro IDs	Signature IDs
8,000	26,227	19,422	36,829

Number of unique features that are present in at least 1 protein in the given organism. For Keywords, it must be present in at least 2 proteins to remove the majority of misspellings.

Figure S3: Pairwise cosine similarity scores between 500 regions for different features.

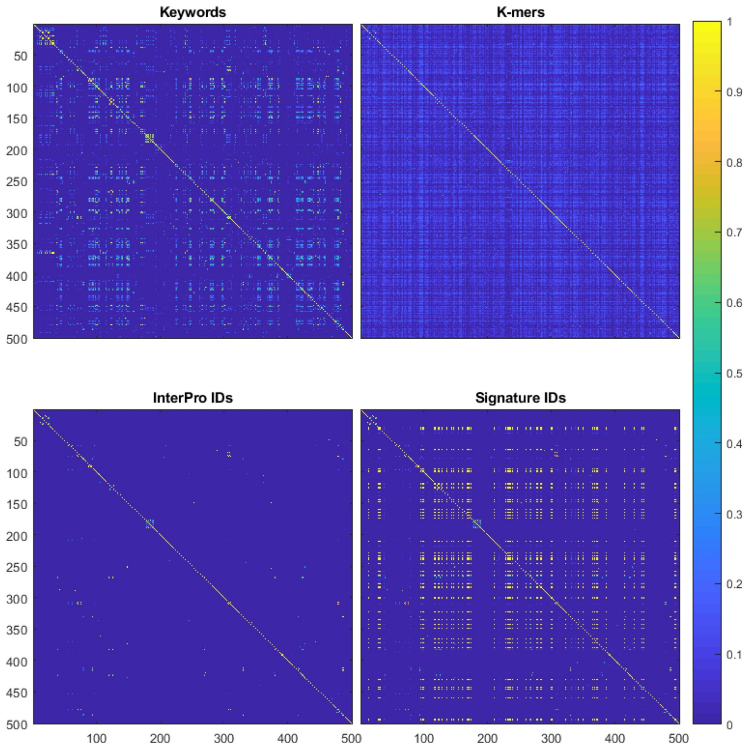

Table S2: Region feature assignment statistics. Number/fraction of regions with and without InterPro domains, and the number/fraction of regions without InterPro domains that have Signature and Keyword features. The mean and median length of these regions are shown on the right.
The same set of statistics are shown below when InterPro families are also included.

Region Assignments	Counts (%)	Region length Mean (median)
**Regions w/ IPR domains**	91,276 (39.7%)	177.1 (137)
**Regions w/o IPR domains**	138,910 (60.3%)	160.4 (87)
W/ Signature ID features	98,776 (71.1%)	194.6 (120)
W/ Keywords features	113,408 (81.6%)	181.1 (107)
**Regions w/ IPR domain + family**	112,275 (48.8%)	195.5 (150)
**Regions w/o IPR domain + family**	117,911 (51.2%)	139.9 (71)
W/ Signature ID features	77,777 (66.0%)	172.6 (96)
W/ Keywords features	92,409 (78.4%)‘	159.6 (86)

W/ = With, W/o = without, IPR = InterPro IDs.

3 Train/validation/test sets

3.1 Region-level evaluation

Table S3: Total number of structurally-verified and semi-manually curated binding region annotations with and without InterPro (IPR) assignments.

Ligands	Total #(pos, neg[3])	With IPR #(pos, neg[3])	W/o IPR #(pos, neg[3])
DNA binding[1]	(524, 188)	(465,105)	(59,83)
RNA binding[1]	(368, 149)	(293,82)	(75,67)
MG binding[2]	(1593, 1125)	(1478,611)	(115,514)
ZN binding[2]	(1907, 910)	(1700,529)	(207,381)

[1] From NBench dataset

[2] From BioLiP dataset

[3] Regions with at least 80% structural coverage but not bound. Nucleic acid binding regions were defined as regions with more than 3 amino acid residues within a cutoff distance of 6 from an nucleic acid molecule in the complex to reduce spurious associations.

3.2 Protein-level evaluation

Train/validation/test sets:

The protein set was divided as follows into training, validation and test sets. See Figure S4 for a visual reference. Root term is excluded as annotation.

- **Training set** - Proteins that had at least 2 annotations at older time-point and did not gain new annotations by new time-point;
- **Validation set** - Proteins that had at least 2 annotation at older time-point and gained at least 1 new annotation by new time-point;
- **Test set** - Proteins that have less than 2 annotations at older time-point but gained at least 2 annotations by new time-point.

After the protein sets have been established, GO terms that fit the following criteria were selected to be tested with the model:

- Have between 30 and 1000 positive annotations in the training set;
- Have at least 30 positive annotations in both the validation and test sets.

These constraints were used to ensure that there is sufficient positive annotations to train and test the model with in order to get meaningful performance reports. The final set of MF-GO terms is 67.

Figure S4: Schematics of temporal holdout data splitting.

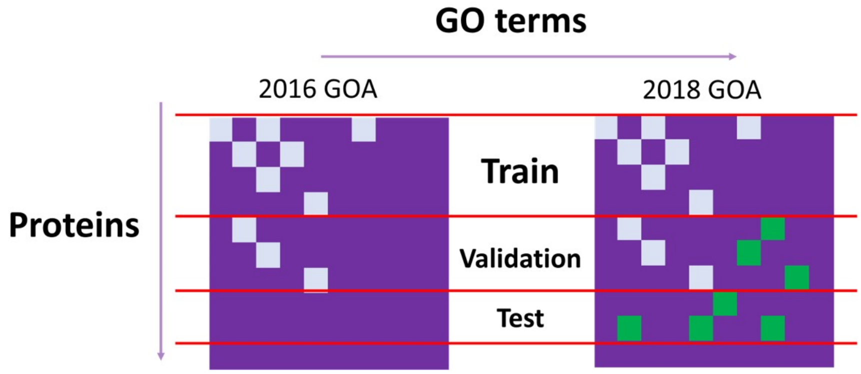

Matrices: Rows represent proteins, columns represent GO terms. Small boxes: GO terms assigned to proteins. Gray = present in the older (e.g. 2016) Gene Ontology Annotation (GOA) database. Green = present in the newer (e.g. 2018) GOA database.

Table S4: Number of train, validation and test proteins used in the protein-level temporal holdout validation of our method.

Total number of MF-GO terms evaluated	67
Total number of SwissProt proteins evaluated	73224
#(train, valid, test)	(39718, 5447, 5746)[1]

[1]22313 out of 73224 reviewed proteins did not fit criteria in any of the defined category.

4 Model hyperparameters

All hyperparameter values are shown in Table S5. We optimize the cost function (**??** in the main manuscript) using mini-batch stochastic gradient descent (SGD) `optimizer` with `momentum`. To prevent overfitting, we implemented `early stopping` by monitoring the performance of the training set after every 50 iterations. We stop the training when the variance of the last `max_variance_length` performance values drops below the minimum variance threshold and after the minimum number of iterations has been completed.

The model is trained with a batch size of at least 128, depending on the number of positive examples for the particular GO term. This is due to the sparsity of positive annotations and the need to have at least one positive example in each mini-batch to calculate the performance value for the stopping criteria. We generated the mini-batches using a stratified sampling strategy to ensure that the class distribution is maintained during training as it appears to perform slightly better overall compared to random sampling in preliminary tests.

We also decreased the learning rate based on the epoch cycle using the given exponential decay formula to improve rate of convergence.

The range of w_1, w_2 and λ values used in the cost function are shown under 'Cost function' and they were narrowed down using a grid search on the region-level evaluation dataset.

Table S5: Model hyperparameters.

Hyperparameters	Values
Stochastic gradient descent	
`batch_size`	$max\left(128, ceiling\left(\frac{\text{number of training examples}}{\text{number of positive examples}}\right)\right)$
`max_epochs`	50
`min_epoch`	3
`lr`	0.1
`lr_decay`	$(1-0.1)^{epoch}$
`momentum`	0.9
`min_variance`	0.00005
`max_variance_length`	15
Cost function	
w_1 `range`	[1e-2 1e0]
w_2 `range`	[0 1e-2 1e0]
λ `range`	[1e-2 1e-1 1e0]

5 Region-level performance

5.1 Bootstrapped performance over all binding regions

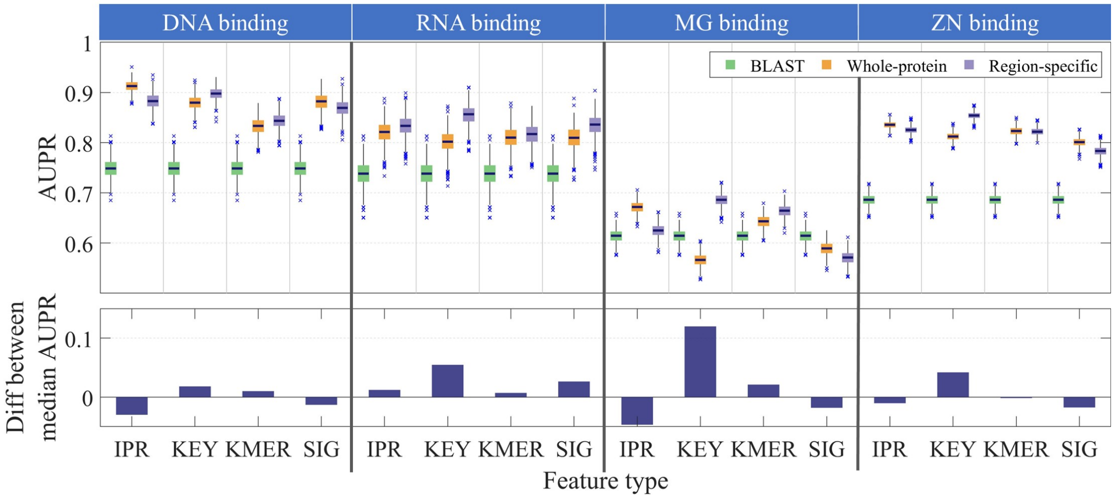

Figure S5: Performance comparisons of region-level predictions using BLAST (green), whole-protein baseline model (orange) versus our region-specific model (purple) for DNA (GO:0003677), RNA (GO:0003723), MG (GO:0000287) and ZN (GO:0008270) binding GO terms.

IPR = InterPro IDs, KEY = Keywords, KMER = K-mers, SIG = Signature IDs. **Upper panels:** Box plots showing the first quartile (Q1), median, third quartile (Q3) and outliers of AUPRs generated over 1000 rounds of bootstrapping.

Bottom panels: Differences between the median AUPR between whole-protein baseline and region-specific methods corresponding to the pair of box plots directly above.

Positive values indicate that the region-specific method outperforms the baseline and vice versa for negative values. Aside from the IPR performances in (b), all differences are significant to at least .001 level based on test statistics from two-tailed Wilcoxon signed rank test.

5.2 Structural examples of predicted binding regions

Table S6: Residue coverage of structures and predicted binding residues.

UniProtKB ID	PDB ID	Chains	Residue coverage of structure	Predicted binding regions
P04386	3coq	A,B,D	8-96	1-49
P01106	1nkp	A,D,B,E	353-434	354-410
O95786	3zd6	A,C,D	230-925	239-452,794-925
P03081	2pf4	E	1-174	76-174
P39286	5uz4	Z	6-339	277-350
Q6N021	5deu	A	1129-1480	1-1289,1290-1905

6 Temporal holdout performance

Figure S6: Performances of protein-level predictions on Molecular Function GO terms on test set using non-IEA annotations only.

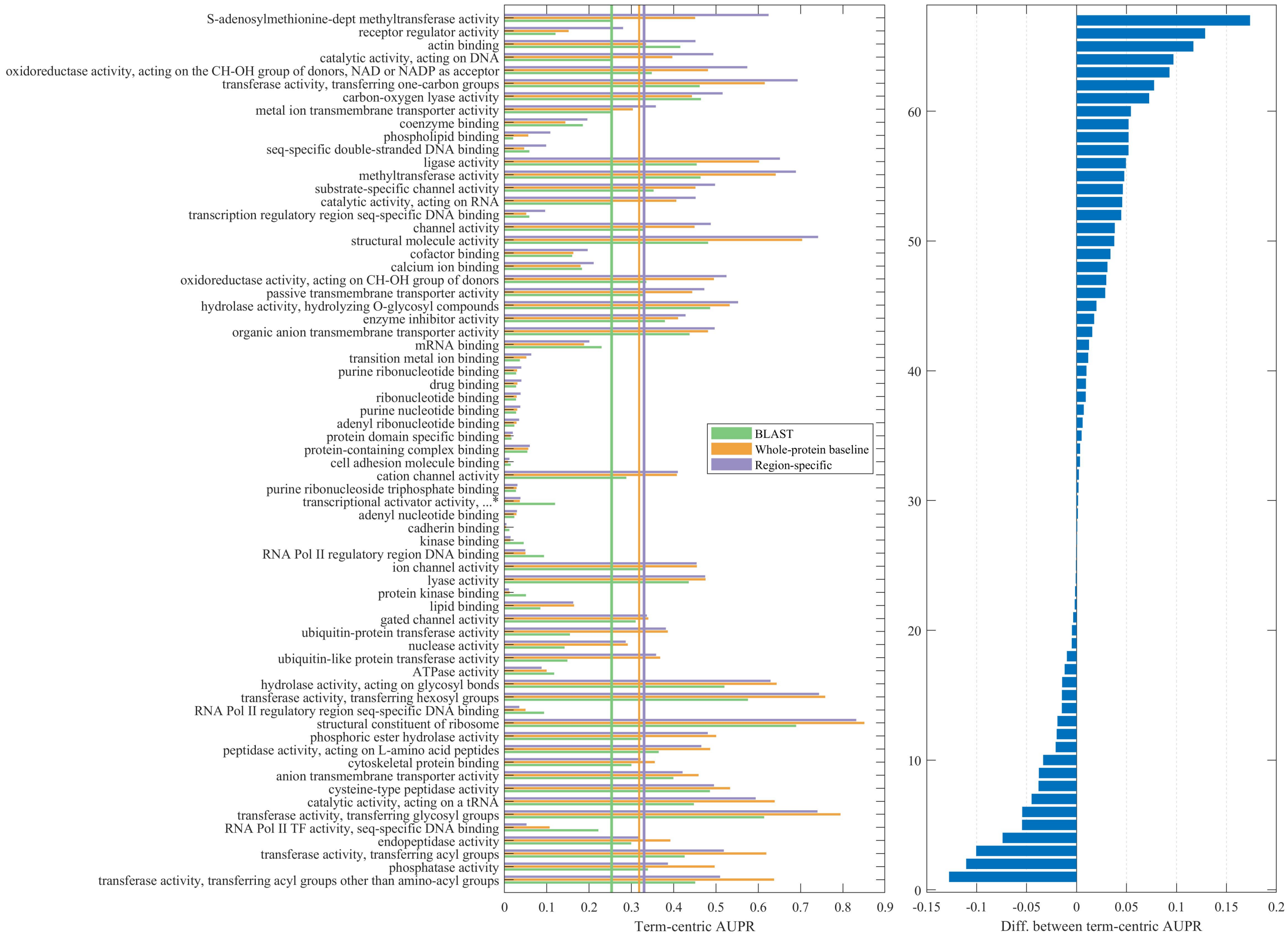

(a) Keyword features

...* = RNA Pol II transcription regulatory region seq-specific DNA binding. Median AUPR scores for the BLAST baseline, whole-protein baseline model and our model for each GO term tested are shown as bar plots in the left panel. Solid line represents the mean over all the GO terms. The GO terms are sorted in descending order of their differences, which are shown in the panel on the right. Positive differences mean that our method performs better than the baseline and negative differences mean the opposite.

Figure S6: Performances of protein-level predictions on Molecular Function GO terms on test set using non-IEA annotations only.

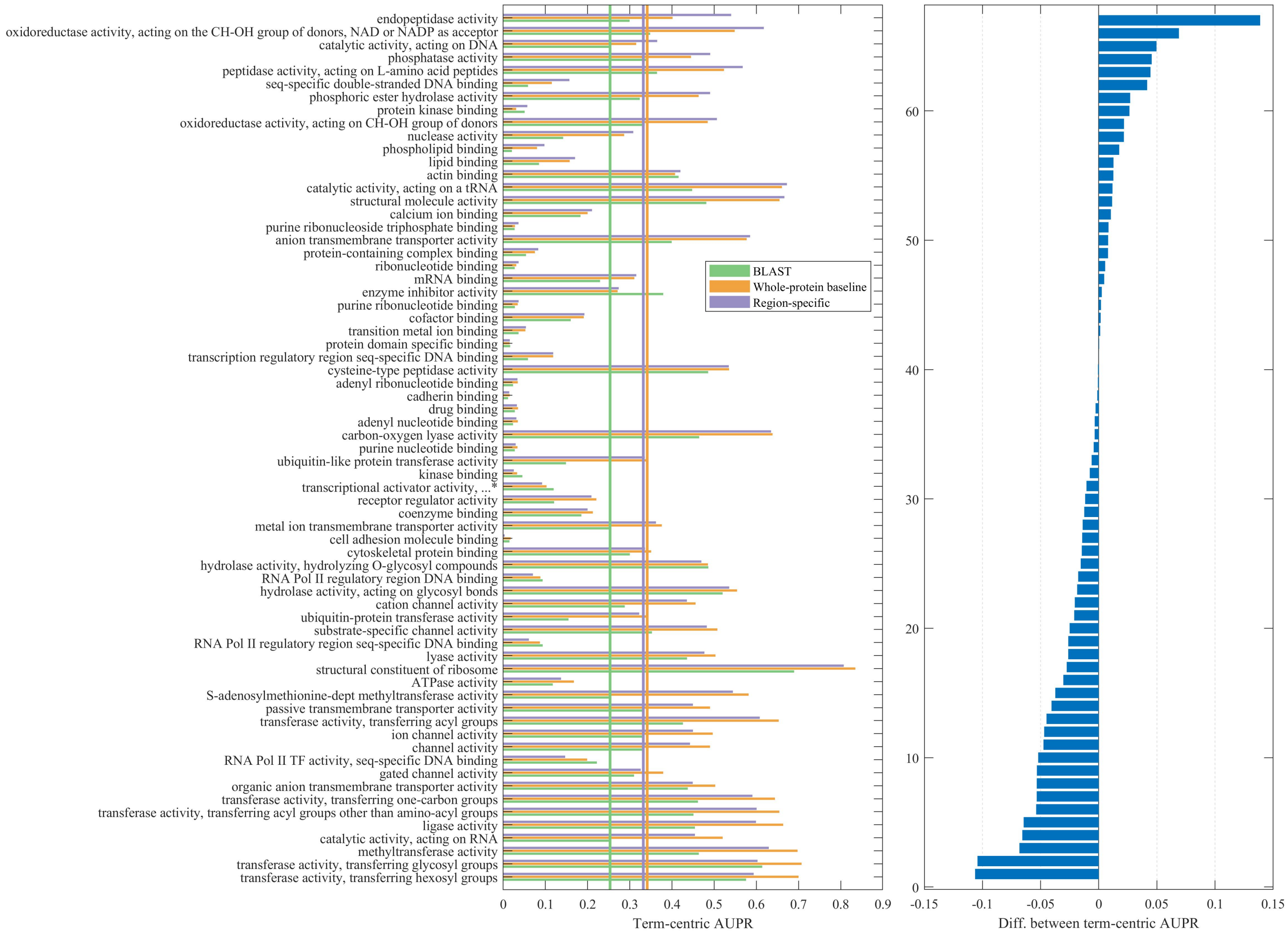

(b) InterPro features

...* = RNA Pol II transcription regulatory region seq-specific DNA binding. Median AUPR scores for the BLAST baseline, whole-protein baseline model and our model for each GO term tested are shown as bar plots in the left panel. Solid line represents the mean over all the GO terms. The GO terms are sorted in descending order of their differences, which are shown in the panel on the right. Positive differences mean that our method performs better than the baseline and negative differences mean the opposite.

Figure S6: Performances of protein-level predictions on Molecular Function GO terms on test set using non-IEA annotations only.

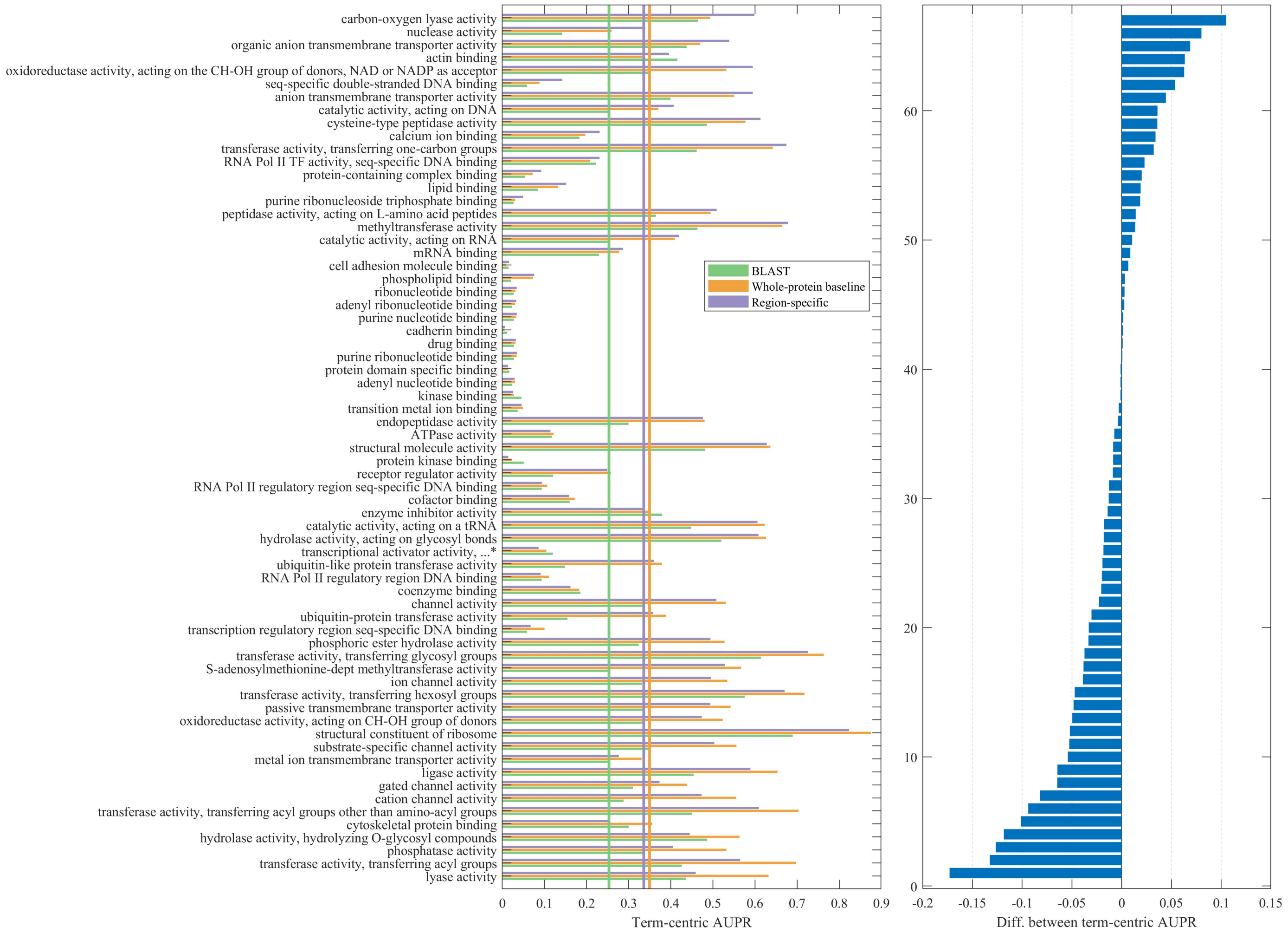

(c) Signature features

...* = RNA Pol II transcription regulatory region seq-specific DNA binding. Median AUPR scores for the BLAST baseline, whole-protein baseline model and our model for each GO term tested are shown as bar plots in the left panel. Solid line represents the mean over all the GO terms. The GO terms are sorted in descending order of their differences, which are shown in the panel on the right. Positive differences mean that our method performs better than the baseline and negative differences mean the opposite.

Figure S6: Performances of protein-level predictions on Molecular Function GO terms on test set using non-IEA annotations only.

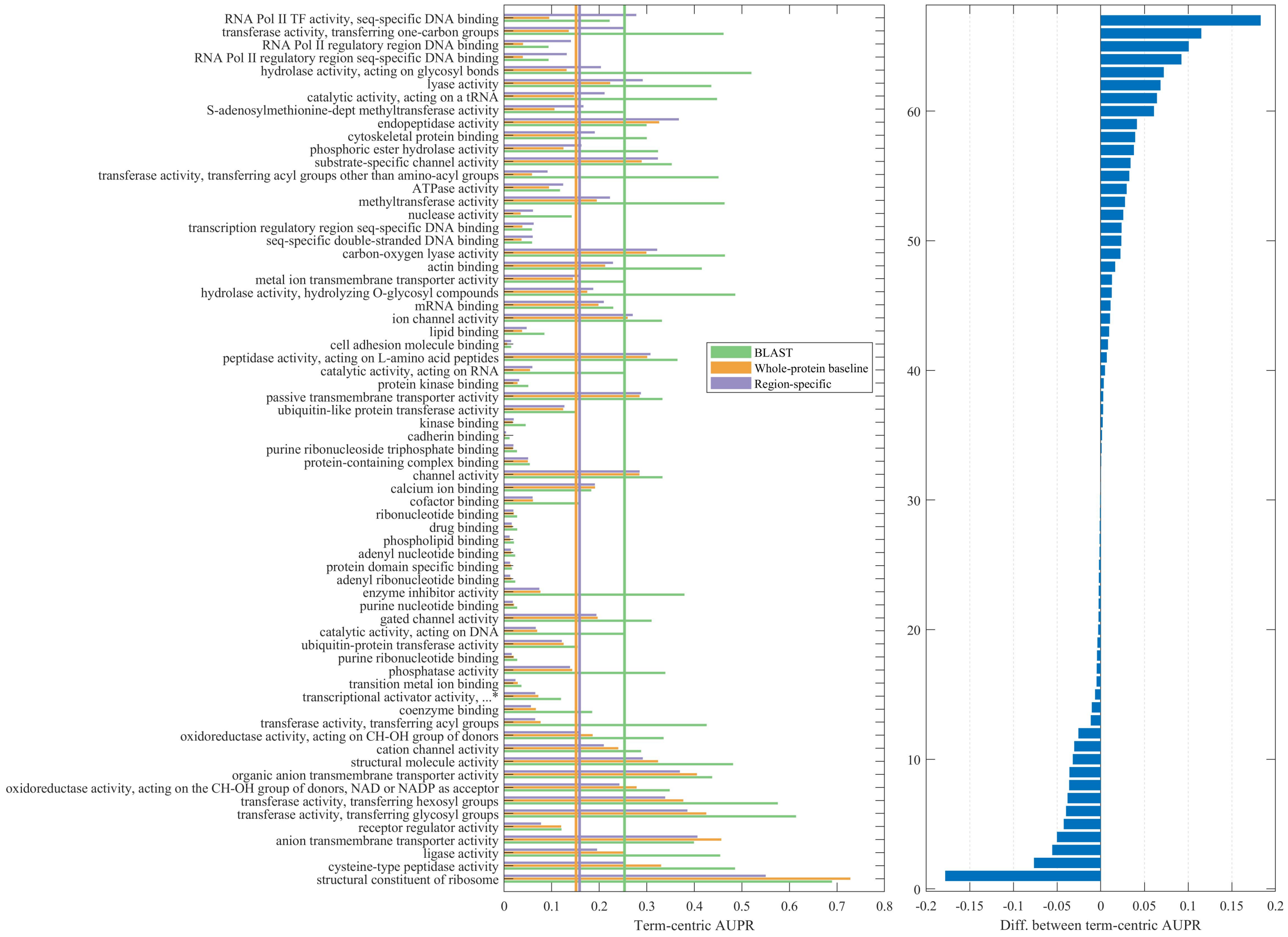

(d) *K*-mer features

...* = RNA Pol II transcription regulatory region seq-specific DNA binding. Median AUPR scores for the BLAST baseline, whole-protein baseline model and our model for each GO term tested are shown as bar plots in the left panel. Solid line represents the mean over all the GO terms. The GO terms are sorted in descending order of their differences, which are shown in the panel on the right. Positive differences mean that our method performs better than the baseline and negative differences mean the opposite.
